# Better Health, Better Lives? 10-Years on From the World Health Organization’s Declaration on the Health of Children With Intellectual Disabilities

**DOI:** 10.1177/17446295221076687

**Published:** 2022-03-09

**Authors:** Nathaniel Scherer, Roger Banks, Melita Murko, Daniel Chisholm

**Affiliations:** International Centre for Evidence in Disability, 4906 London School of Hygiene & Tropical Medicine, London, UK; Learning Disability and Autism Programme, NHS England, London, UK; Mental Health Unit, 93773 World Health Organization Regional Office for Europe, Copenhagen, Denmark

**Keywords:** intellectual disabilities, child, health, Europe, review

## Abstract

It is now 10 years since the *European Declaration on the Health of Children and Young People with Intellectual Disabilities and their Families: Better Health – Better Lives* was adopted by the World Health Organization. Through discussions with key informants and an online literature review, we reflect on actions and progress made in line with this Declaration to improve the health and wellbeing of children with intellectual disabilities and their families. Despite finding positive examples of policy, legislation and practice in support of children with intellectual disabilities, there are clear gaps and areas for improvement. Countries must continue to take action, as supported by the World Health Organization and other such organisations, in order to support children with intellectual disabilities in realising their fundamental human rights.

## Background

In 2010, the World Health Organization Regional Committee for Europe endorsed the *European Declaration on the Health of Children and Young People with Intellectual Disabilities and their Families: Better Health – Better Lives* (World Health Organization, 2010). Recognising Article 25 of the United Nations *Convention on the Rights of Persons with Disabilities*, attuning ‘the highest attainable standard of health without discrimination on the basis of disability’, this Declaration encouraged World Health Organization Member States across Europe to support the health and wellbeing of children with intellectual disabilities and their families, advocating direct reform to health care systems and the wider social protection environment, including deinstitutionalisation (United Nations, 2006).

The Declaration, formerly Resolution *EUR/RC61/R5*, identified 10 priorities for action, in support of change:

1. Protect children and young people with intellectual disabilities from harm and abuse.
2. Enable children and young people to grow up in a family environment.
3. Transfer care from institutions to the community.
4. Identify the needs of each child and young person.
5. Ensure that good quality mental and physical health care is coordinated and sustained.
6. Safeguard the health and wellbeing of family carers.
7. Empower children and young people with intellectual disabilities to contribute to decision-making about their lives.
8. Build workforce capacity and commitment.
9. Collect essential information about needs and services and assure service quality.
10. Invest to provide equal opportunities and achieve the best outcomes.

The Declaration was developed in collaboration with international experts in the field of the intellectual disability, and each of these priorities for action was supported by an expert technical paper, including an article focussed on areas for future research.

In 2016, a World Health Organization mid-term report noted, among others, the following examples of progress in line with the priorities of the Declaration: *Investing in Children: the European Child and Adolescent Health Strategy 2015–2020 (EUR/RC64/12)*, which included mental disorders and disabilities in young people (World Health Organization, 2014a); and *Investing in Children: the European Child Maltreatment Prevention Action Plan 2015–2020 (EUR/RC64/13)*, which identified that children with disabilities or behavioural problems and those in institutional care may be at increased risk of maltreatment (World Health Organization, 2014b). In the same year, the World Health Assembly adopted Resolution *WHA67.8 - Comprehensive and coordinated efforts for the management of autism spectrum disorders,* requesting Member States to give appropriate recognition to the needs of individuals with autism spectrum disorders and other developmental disabilities (World Health Assembly, 2014). The 2016 mid-term report stressed the importance of continued working relations with UNICEF in achieving Declaration objectives, as well as with the European Commission on deinstitutionalisation and with the United Nations Office of the High Commissioner for Human Rights (OHCHR) on human rights in institutions (World Health Organization, 2021a).

Ten years on, as a final report on progress against the Declaration and associated action plan is submitted to the World Health Organization Regional Committee for Europe, we reflect on the impact this Declaration has made and where countries across Europe go next.

## Methodology

Data was collected in June 2020, primarily through a key informant methodology. Key informants from across Europe were identified through existing networks, previous contributors to the Declaration and additional snowballing methodologies, along with online searches of organisations working in this area, both at a national and local level. Researchers sought key informants from each country falling under the World Health Organization European Region, and from cross-continental groups and organisations.

In total, 15 key informants were recruited. 12 respondents were from, and gave information pertinent to, Austria, Bosnia and Herzegovina, Czechia, Estonia, Finland, Germany, Georgia, Latvia, Italy, Malta, Switzerland and the United Kingdom, which presented a geographic spread across Europe and country income-level. Three respondents worked for third sector organisations working across Europe, supporting and advocating for children with intellectual disabilities. Using a standardised form, each key informant provided information on progress against the priority actions outlined in the Declaration within their country or region of expertise. Information was requested on actions and gaps relevant to: (1) Policy; (2) Legislation; (3) Professional Development; (4) Projects/Programmes; (5) Research; (6) Other.

Findings from these key informants were supplemented with a literature review of actions taken across Europe in line with the Declaration’s priorities, with particular focus paid to regions and countries not captured by the key informants. The literature review was conducted in Google Scholar and the grey literature database OpenGrey using key terms associated with the declaration: intellectual disability; child; health. In addition, we conducted follow-up interviews with respondents from the cross-continental third sector organisations and interviews with members of the original steering group for the Declaration. In these interviews, we discussed the key themes emerging and priority recommendations for the next 10 years.

Information provided by the key informants and the literature review have been narratively synthesised into the central themes seen across Europe. We have matched each of these themes against the priority actions of the Declaration and provided specific country examples, where relevant.

## Findings

### Equal Opportunities (Priority Actions: 4, 7, 10)

People with intellectual disabilities are not equal members in society. They are less likely to work, less likely to achieve a full education and more likely to live in poverty. The Declaration called for this injustice and inequality to end, and many countries across Europe have sought to address inequalities in opportunity experienced by persons with disabilities, with evidence of policy development and reform since 2010. In Czechia, for example, the *National Plan for the Promotion of Equal Opportunities for Persons with Disabilities* was released in 2015, covering strategic areas in support of people with disabilities, including equal treatment and discrimination, public education and health care (Government of the Czech Republic, 2015). However, key informants reported that much of the progress made in policy reform has focused on adults with disabilities, rather than specific actions for children. Where children are included or focused upon, children with intellectual disabilities are often ignored, with a greater emphasis placed on support for children with physical and sensory impairments. Reasons reported for this neglect centred on stigma towards children with intellectual disabilities and their families, as well as a lack of information, training and knowledge on accessibility needs and reasonable adjustments. Many service providers (whether that be in health care, education or social care) were reported to have a greater understanding of what constitutes an inclusive environment for children with physical and sensory impairments than they would for those with intellectual disabilities.

As recognised in Priority Action 7 of the 2010 Declaration, one way in which to combat inequalities in opportunity is by empowering children with intellectual disabilities to contribute to the decisions affecting their lives. In 2017, Save the Children Norway facilitated a workshop with children with intellectual disabilities, in which they contributed their experiences and advice towards the implementation of rights for this group (Save the Children Norway, 2019). These opinions were included in reporting towards the United Nations *Convention on the Rights of the Child* and have been used to advocate change with Norway’s National Government. In Russia, Perspektiva organise a yearly international disability film festival, *Breaking Down Barriers* (Perspektiva, 2020). Founded in 2002, this festival aims to increase the voice of people with disabilities, and reduce stigma and discrimination. Children with and without disabilities vote on the best children’s film and there is a category for best film on intellectual disability. More widely implemented across Europe continues the project *Hear Our Voices!* coordinated by Inclusion Europe, in which partners developed mechanisms to support children with intellectual disabilities to participate in their communities, including decision-making processes (Inclusion Europe, 2014). Despite these examples, many key informants reported little to no action in empowering children with intellectual disabilities and their families in decision-making processes in their country and across Europe. Informants reported a lack of participation and representation of people with intellectual disabilities and their families in local, national and regional planning and decision-making. As well as limiting the effectiveness of proposed projects and policies, this lack of encouraged participation places a significant burden on families, who have to continually ‘fight’ to be included in the planning process, on behalf of themselves and their children.

Between 2011 and 13, the *Turning Words into Action* project, led by LUMOS, brought together children and young people with intellectual disabilities, their parents, policy makers, and health and educational professionals in Bulgaria, Czechia and Serbia, with the aim of bringing the *Better Health, Better Lives* Declaration to life through meaningful and effective child participation activities and outcomes (Lumos, 2014). The project, funded by the European Commission Social Innovation Fund, supported young people to become spokespeople, giving them the opportunity to influence and advise on how to implement the Declaration in their own countries. It also, for the first time, provided these young people with the opportunity to interact with policy makers and discuss issues around their disability as equal partners. In this way, the goal of the project falls in line with the Declaration’s target to achieve real impact, demonstrating a programme that can be used to improve governments’ and societies’ approach to caring for children with intellectual disabilities and providing a template for other countries to follow.

### Safety and Safeguarding (Priority Actions: 1, 3, 6, 7)

In order to live a healthy and happy life, all children need to live in an environment safe from harm and abuse. Children with intellectual disabilities are particularly vulnerable to abuse, with evidence showing this to be particularly true if living in an institution. In 2015, the European Union Agency for Fundamental Rights developed a comprehensive report on *Violence against children with disabilities: legislation, policies and programmes in the EU* (European Union Agency for Fundamental Rights, 2015). European Union Member States reported that those with intellectual disabilities were more likely to be bullied and abused than those with more ‘visible’ physical disabilities. Respondents also reported that abuse against children who communicate in a non-traditional way was less likely to be prosecuted. The report outlines a number of policies, legislative and programmatic approaches to addressing issues of safeguarding across Europe. As reported, in 2015 a number of Member States held policies addressing the rights of persons with disabilities and their protection from violence: Austria, Czechia, Finland, Germany, Italy, Portugal, Slovenia and Spain. However, a number of policies fail to provide specific objectives and action points for children with disabilities. In Finland and Portugal, for example, policies recognise the increased risk of violence for children with disabilities, but fail to establish specific measures for addressing or preventing such violence. Examples of support in other areas include capacity building and awareness raising campaigns, such as the programme *My Body is My Own* from the Hand in Hand Foundation in Hungary, in which children (and adults) with intellectual disabilities receive training in sex education, situational awareness and self-defence (Hand in Hand Foundation, 2021). In Sweden, the organisation TRIS has developed education material entitled *Triple Victimised*, providing information on honour-related oppression and violence against young people with intellectual disabilities (TRIS, 2021), and in Italy, students can take a Diploma course in safeguarding at the Pontifical Gregorian University, which includes a lecture on the protection of children with intellectual disabilities from abuse, and an additional elective module (Pontifical Gregorian University, 2021).

Progress is being made (although it is important to consider the European Union Agency for Fundamental Rights report is from 2015), but challenges remain, including: a lack of specialised support for children with intellectual disabilities; limited knowledge and awareness of protection and prevention measures; lack of family support; and insufficient professional capacity and funds. The European Union Agency for Fundamental Rights recommends improvements from European States in the following areas, which parallel closely the priorities of the World Health Organization Declaration:

• More inclusive child protection systems
• Enhanced legal and political frameworks
• Improved service coordination
• Addressing societal attitudes
• Promoting child-focused prevention and child participation
• Providing family-focused services
• Ensuring inclusive education
• Advancing deinstitutionalisation
• Developing targeted tools, allocating resources and improving human resource capacity
• Collecting data

Regular and coordinated cooperation across these areas is needed among multilateral organisations, European Member States, national service providers, local authorities, experts and people with lived experience, in order to identify risk situations and address the vulnerability of children with intellectual disabilities to abuse and violence.

### Community-care (priority actions: 2, 3, 4, 5)

Children with intellectual disabilities undoubtedly benefit from a stable and nurturing family environment (Olusanya et al., 2018). Nurturing care is key to a child’s development, helping them reach their potential in all areas of life, including good physical and mental health (Britto et al., 2017). Early childhood interventions are the subject of wide attention, focussing on reducing the impact of factors associated with developmental disability, supporting behavioural challenges and improving parent-child interactions. The 2018 World Health Organization publication, *Nurturing Care for Early Childhood Development*, proposes an evidence-based framework and ‘roadmap for action’ to bring together parents and caregivers, national governments, civil society groups, academics, the United Nations, the private sector, educational institutions and service providers in ensuring the best start in life for all children (World Health Organization, UNICEF and World Bank, 2018). However, children with intellectual disabilities in many countries have often been separated from their families and live in large residential institutions that cannot meet their needs (UNICEF, 2013). In 2015, an estimated 1,000,000 children lived in institutions of this kind across Europe (Desmond et al., 2020), and according to UNICEF, across Eastern Europe and Central Asia, children with disabilities are almost seventeen times more likely than other children to be institutionalised (UNICEF, 2012). In some instances, parents may be encouraged by medical professionals and the wider community to abandon their ‘sick’ child to the care of institutions, as is reported to be a regular occurrence for parents of children with Down syndrome in Central Asia and post-Soviet states (Child Rights International Network, 2014; Open Society Foundations, 2018; Tearfund, 2019).

From 2013 to 2019, Eurochild coordinated the *Opening Doors for Europe’s Children Campaign*, aiming to strengthen families and end institutionalisation of children in Europe. A recent end-line report showed that several countries across Europe have continued to put in place the legislative and policy frameworks necessary to achieve a systemic and sustainable reform of their care system (Eurochild, 2020). Child protection reforms are currently being implemented in Bulgaria, Bosnia and Herzegovina, Croatia, Estonia, Latvia, Lithuania, Moldova, Romania and Ukraine, with the support of national strategic frameworks on deinstitutionalisation. In addition, a number of alternatives to institutionalisation have been increasingly promoted. For instance, legislation on foster care and on the protection of families with children were adopted in Bosnia and Herzegovina in 2016, Belgium in 2017, and Greece and Croatia in 2018 (Eurochild, 2016; Eurochild, 2017; Eurochild, 2018b; Eurochild, 2018a). Despite evidence of progress, the report details instances where the system does not function optimally, disrupting the positive trend towards development of foster care and other social care systems. In 2018, National Coordinators in Croatia, Hungary, Moldova and Ukraine reported that support services for foster families are rare or insufficient and often do not cover a child’s basic needs (Eurochild, 2020). Unavailable and deficient provision of specialised foster care for children under the age of three and for children with disabilities were also reported. In a separate 2020 report, 25 of the 27 European countries appraised still had in use large institutional settings for children with disabilities, although there has been progress made towards deinstitutionalisation (Šiška and Beadle-Brown, 2020). Since 2009, 11 of the countries included in the report saw a decrease in the use of institutions for children with disabilities. In many cases, these children moved back to live with their families, or were fostered or adopted. However, two countries saw an increase in the use of institutions over this period and four saw no change. The remaining eight did not have data available. Children with intellectual disabilities, autism and those who present behavioural challenges are reported to be the most likely groups to be living in large residential institutions, and in countries where large institutions are no longer in use, adults and children with intellectual disabilities are least likely to benefit from personal assistance, and are more likely to be living in local residential care, such as group homes. Although there appears to be progress in deinstitutionalisation, transitioning children with intellectual disabilities to community-care, the extent of this progress is varied and slow in many countries. In reflection, very few key informants in this study reported progress in deinstitutionalisation, with many actively citing this area as a major challenge to be addressed going forwards.

Key informants reported a number of common themes from across Europe which hinder the transition of children to community life. Firstly, there is a reported trend of transferring responsibility for target groups (including children with intellectual disabilities) from national or federal level to regional level. This transfer has caused issues in budget allocation, effective coordination and competence of services. The child protection sector continues to be underfinanced and lacks the capacity to ensure the transformation of social services, sustaining a reliance on institutions. There is seemingly a shortage of staff, poor material resources and high turnover of professionals who lack training and supervision to change practice in child protection. Secondly, although policies and strategies have been developed in many countries, these have often failed to provide details on implementation and monitoring. Very little focus is placed on long-term sustainability and how to scale up results. The focus appears to be on there being an ‘EU funded project’, with no plans in place for continued implementation once funds cease to be available. Finally, there seems to be a disconnect between the definition of community-care stated in the United Nations *Convention on the Rights of Persons with Disabilities* and what is delivered in practice. There are examples of smaller institutions being set up or older, larger institutions simply being split into smaller units, with no material difference in the nature of, or attitude towards, care of children with intellectual disabilities. For instance, an informant gave the example of a situation where children with disabilities are enrolled in special boarding schools during the week and then transferred at the weekend to other institutions.

It is apparent that the transition of children with intellectual disabilities to community-care and family life remains a priority. Progress requires consistent buy-in and understanding of what this means across Europe, with action plans and implementation strategies in place to guide national governments and local authorities. Limited data continues to be a constraint, and local, national and continental research and reporting remains key in understanding the lived experiences of children with intellectual disabilities before, during and after the transition to community-care. Lessons learned on ‘what works’ need to be disseminated across Member States. Ultimately, instead of funding institutions, funding needs to focus on establishing more community-based support. Accessible early intervention and support can help to prevent institutionalisation of children, and should be provided in all European countries.

### Health and Social Care (priority actions area: 5, 6, 8, 9)

Although there are inevitable variations across country, health and social care systems, and health and social care economies, evidence shows that children with intellectual disabilities experience higher mortality, morbidity and health care inequalities than children without intellectual disabilities (Bourke et al., 2017; Emerson and Spencer, 2015; Olusanya and Nair, 2019). In the priority actions of the 2010 Declaration, there are a number of identified elements that contribute to effective health care for children with intellectual disabilities: identifying a child’s needs; building workforce capacity; and ensuring good quality physical and mental health service provision. To support the Declaration and initiatives across the continent, the World Health Organization Regional Committee for Europe endorsed Resolution EUR/RC64/12, *Investing in children: the European child and adolescent health strategy 2015–2020*, which incorporates support for children with disabilities (World Health Organization, 2014a). The final progress report (published in September 2020) does not make any mention of disability specifically, although progress made for all children across the World Health Organization European Region is outlined; this includes information on rights and participation, mental health and access to health services (World Health Organization, 2020a).

Developed out of Resolution EUR/RC64/12 comes the 2020 *Framework on Early Childhood Development in the World Health Organization European Region*, which provides countries with guidance on helping children to reach their full potential (World Health Organization, 2020b). Actions and guidance is given for the support of children with disabilities and developmental disorders, including children with intellectual disabilities. Guidance includes information relevant to: risk to childhood development; classification systems of disability; inclusion and discrimination; and supportive health systems. For example, the framework addresses the use of non-evidence-based approaches with children with intellectual disabilities, and suggests actions to integrate evidence-based strategies and address other harmful practices in health systems.

In a promising example of support to child health, Special Olympics have been extending events and hosting programmes for children with intellectual disabilities across the region, who may never have participated in sport, supporting their health, wellbeing and inclusion. Programmes such as *Young Athletes* and *Unified Sports* have been implemented widely, from Germany to Uzbekistan, with research demonstrating improvements in psychosocial wellbeing, inclusion, physical health and attitudes of others (Hassan et al., 2012; Myśliwiec and Damentko, 2015; Özer et al., 2012). The Special Olympics World Winter Games will be held in Kazan, Russia in 2023, with plans for widespread youth engagement.

Despite such promising programmes, information on health outcomes and health care provision for children with intellectual disabilities is generally limited, with key informants reporting a lack of reliable data, and children and adolescents with intellectual disabilities (and children with disabilities more broadly) often neglected in studies and national statistics. Those figures that do exist are typically out-of-date, not disaggregated and not comparable with international data. Informants called for more research, adopting clear participatory approaches. Limited data and information is a key barrier to the effective planning and provision of coordinated and sustained health care for children with intellectual disabilities. Lessons can be learned from the *Learning Disabilities Mortality Review (LeDeR) Programme* in England (UK), the world’s largest study and database on the causes of death in people with intellectual disabilities (University of Bristol, 2020). Collecting good quality data ensures accountability and supports advocacy, whilst providing evidence on the long-term effectiveness of health care initiatives.

As well as improvements in data collection, initiatives are needed to enhance professional skills and capacity, health and welfare services, and decision-making in the health care process. Examples of such initiatives are in evidence across Europe, such as the World Health Organization *Caregiver Skills Training* programme for families of children with developmental delay, as recommended in the *mhGAP Intervention Guide* (World Health Organization, 2021b). In 2019, the International Centre for Evidence in Disability at the London School of Hygiene & Tropical Medicine developed a 3-week online course *Integrated Healthcare for Children with Developmental Disabilities*, with the target audience being health care professionals across the world looking to improve the health and wellbeing, and health care provision, for children with developmental disabilities (including intellectual disability). Close to 8,000 people have enrolled on this course (International Centre for Evidence in Disability, 2019). In Estonia, the programme *Development and Provision of Support Services for Disabled Children* was carried out from 2014-2020. The project, funded by the European Social Fund, sought to develop and provide support services to children with severe disability, including affordable child care and transport services to health and rehabilitation institutions (Republic of Estonia, 2014).

Improvements in health and social care are interdependent on other areas of government policy, such as education and welfare (housing, for example). In England, *Building the Right Support* is the national plan for children and adults with learning (intellectual) disabilities and with autism, developed between national health and social care agencies, together with input from people who use services and their families (NHS England, 2015). This plan looks to transform care partnerships and foster greater cooperation across service providers. Examples of this kind were not, however, seen across Europe, and key informants did not generally report clear, nationally driven strategies and policies in this regard, in their respective countries.

In general, many informants noted little to no progress made in health and social care specifically, and what progress has been made has typically focused on adults with intellectual disabilities, with children left behind. Greater emphasis is needed in health and social care across Member States if progress is to be seen and sustained.

As well as child health, it is important to recognise the ongoing support needed for family members and caregivers. Although we should not ignore the positive outcomes of raising a child with intellectual disabilities, evidence from across Europe and other regions demonstrates the negative impact on many family members. A 2019 systematic review and meta-analysis showed that 31% of parents of children with intellectual and developmental disabilities have depressive symptoms, compared to 7% of parents of children without intellectual and developmental disabilities (Scherer et al., 2019). Stress is associated with caregivers’ poorer mental health and wellbeing, and parents have reported feelings of emptiness, loneliness and rejection (Cramm and Nieboer, 2011; Di Giulio et al., 2014). Caregivers need support to reduce the burden and stress of caregiving, and to promote mental wellbeing. The World Health Organization has developed the *Caregiver Skills Training Program for Developmental Disorders or Delays*, for use in low- and middle-income countries, which lies within the *mhGAP Intervention Guide* (Salomone et al., 2019). This programme supports caregivers to promote a child’s learning, social communication and adaptative behaviour, and also aims to increase the skills and confidence of caregivers, helping to reduce stress and improve their wellbeing. We could not find evidence into the effectiveness of this programme on improving the health and wellbeing of caregivers in Europe, and this would be a useful area to explore in future research. Overall, evidence on caregiver support in Europe was limited, both in the literature review and coming from key informants. A 2013 systematic review found perceived benefit of peer-support groups among parents of children with disabilities, but of the 17 papers included, only three were conducted in Europe and all of these were from the UK (Shilling et al., 2013). Further evidence into such support programmes is needed across the World Health Organization European Region to inform health care delivery for a group experiencing negative impacts to their psychological health and wellbeing.

## Discussion and Next Steps

Although progress has been made across Europe to better the health and wellbeing of children with intellectual disabilities, they remain a group left behind in policy, legislation and practice. We have demonstrated some degree of progress against the priorities of the 2010 Declaration, with a number valuable and interesting examples of policy and programmes, but there remain inconsistencies, gaps and areas to address.

Data coming out of the COVID-19 pandemic is highlighting the extent to which people with disabilities, and in particular people with intellectual disabilities, are continually forgotten and abandoned in national policy, strategy, and in this instance, response to crisis (Amor et al., 2021; Constantino et al., 2020; Doody and Keenan, 2021; European Association of Service Providers for Persons with Disabilities, 2020; Shakespeare et al., 2021a, 2021b; Willner et al., 2020). Despite, in this crisis, people with intellectual disabilities being one of the groups most vulnerable to death and serious illness, they continue to be abandoned and treated as second-class citizens (Williamson et al., 2021). The pandemic has not created new problems for people with intellectual disabilities; it has simply brought to the fore that which already existed. There is a plethora of evidence as to the challenges these individuals face: violence, abuse, poverty, isolation, loneliness and access to education, employment and health care (Adugna et al., 2020; Ali et al., 2013; Banks et al., 2017; Bowen and Swift, 2017; Emerson and Spencer, 2015; Jones et al., 2012; Kuper and Heydt, 2019; McGarty and Melville, 2018; Mitter et al., 2018; Petroutsou et al., 2018; Tomsa et al., 2021; Webster and Carter, 2007; Williamson et al., 2021). That cited is but a drop in the ocean of data available to us on the challenges and difficulties and unacceptable situations adults and children with intellectual disabilities live with day to day. Simply, our society does not support children with intellectual disabilities to achieve the same quality of life as every citizen and to realise their basic human rights. Children and adults with intellectual disabilities are often invisible in society and this makes it easy for them to be overlooked and marginalised when it comes to policy decisions and funding. This must change.

Guidance on the next steps must be provided by multilateral agencies, such as the World Health Organization and the United Nations, with support from disability and development organisations, who have expertise in realising change for people with intellectual disabilities. The United Nations *Convention on the Rights of Persons with Disabilities* has been in effect for over a decade, and nations must keep momentum to ensure equal and fair societies that embrace people with disabilities and children with intellectual disabilities as valued members. In doing this, they must be held accountable to highest moral, social and practical standards, lest adults and children with intellectual disabilities continue to be left behind.

In making progress in line with the *Convention on the Rights of Persons with Disabilities*, countries must place adults and children with intellectual disabilities and their families at the forefront of decision-making processes. This means meaningful consultation. This means putting people with lived experience in positions of power. And it means funding and raising up the status of organisations of persons with disabilities (OPDs) and other disability advocacy groups. People with lived experience and organisations of and for people with disabilities know what is best for them and their community. Still, today, consultation is too often cursory, in line with a tick-box exercise only. Policymakers and practitioners must realise that they make decisions that have far reaching consequences, and only by putting power into the hands of the communities impacted will change be appropriate, sustainable and meeting real need.

Based on our findings and conversations with our key informants, we outline below a number of considerations and steps that countries and multilateral organisations could take to ensure and expedite continued progress across Europe:

1. Continue and enhance awareness raising on the developmental, emotional and social needs of children with intellectual disabilities, as well as information on how to develop an inclusive environment. This knowledge is needed for national and local government, service providers, and public and private sector organisations, as well as the general public.
2. Increase empowerment, participation and representation of children and adults with intellectual disabilities and their families in planning and decision-making at individual, local and national levels. This includes empowering people with intellectual disabilities and their families into leadership positions, and holding OPDs as equal partners.
3. Promote and fund regional and national actions to address health inequalities and premature morbidity and mortality in individuals with intellectual disabilities and their families. This should involve addressing the social determinants that contribute to poorer health among children with intellectual disabilities, such as stigma and violence.
4. Mainstream health and social care reform in strategies and policies, ensuring each is provided with clear details on implementation and monitoring, with support provided at national level.
5. Continue progress towards deinstitutionalisation, supported by a clear and consistent understanding of what constitutes community-care and effective community-based interventions.
6. Improve research, information gathering and monitoring of progress across countries, with accountability structures for Member States to the World Health Organization. As an example, this would include accountability against the explicit inclusion of indicators agreed by Member States in the envisaged *Implementation and monitoring framework for mental health promotion, protection and care in the World Health Organization European Region for 2021–2030.*

These actions are not a wish list. They are absolutes for all nations that want to see children with intellectual disabilities and their families realise their human rights. Progress and action are needed and needed now.
